# More or less: The selection of indel in the 3′UTR of *ZMET2* modules genome methylation and husk layer number in maize

**DOI:** 10.1093/plphys/kiae182

**Published:** 2024-03-28

**Authors:** Yee-Shan Ku

**Affiliations:** Assistant Features Editor, Plant Physiology, American Society of Plant Biologists; School of Life Sciences and Centre for Soybean Research of the State Key Laboratory of Agrobiotechnology, The Chinese University of Hong Kong, Hong Kong SAR, China

Maize husk is an important structure protecting the inner ear from heating, drying, pest damage, and pathogen infection (Wang et al. 2023). The typical maize husk number ranges from 7 to 15 (Cui et al. 2016). Tropical or subtropical varieties usually have more husk layers than temperate varieties (Cui et al. 2016). Despite the protective advantages, too many husk layers limit kernel dehydration rate and impede harvest (Wang and Li 2017; Li et al. 2018; Zhou et al. 2020). Epigenetic regulations provide tunable strategies to shape crop traits for environmental adaptation (Lieberman-lazarovich et al. 2022). However, the role of epigenetics in regulating husk layer number in maize has remained largely unexplored.

In this issue of *Plant Physiology*, Wang et al. report the modulation of maize husk layer number by *ZMET2*, which encodes a DNA methyltransferase (Wang et al. 2024). Using more than 500 maize inbred lines, the authors studied the association between various agronomic traits and DNA methylation-related genes, including those encoding DNA methyltransferase and demethylase (Wang et al. 2024). Among the 19 agronomic traits and the 11 DNA methylation related genes, only 3 single-nucleotide polymorphisms within *ZMET2* were significantly associated with the number of husk layers (Wang et al. 2024). Further sequence analyses showed that maize lines without a 10-bp insertion in the 3′ UTR had fewer husk layers than those with the insertion (Wang et al. 2024).

**Figure 1.**
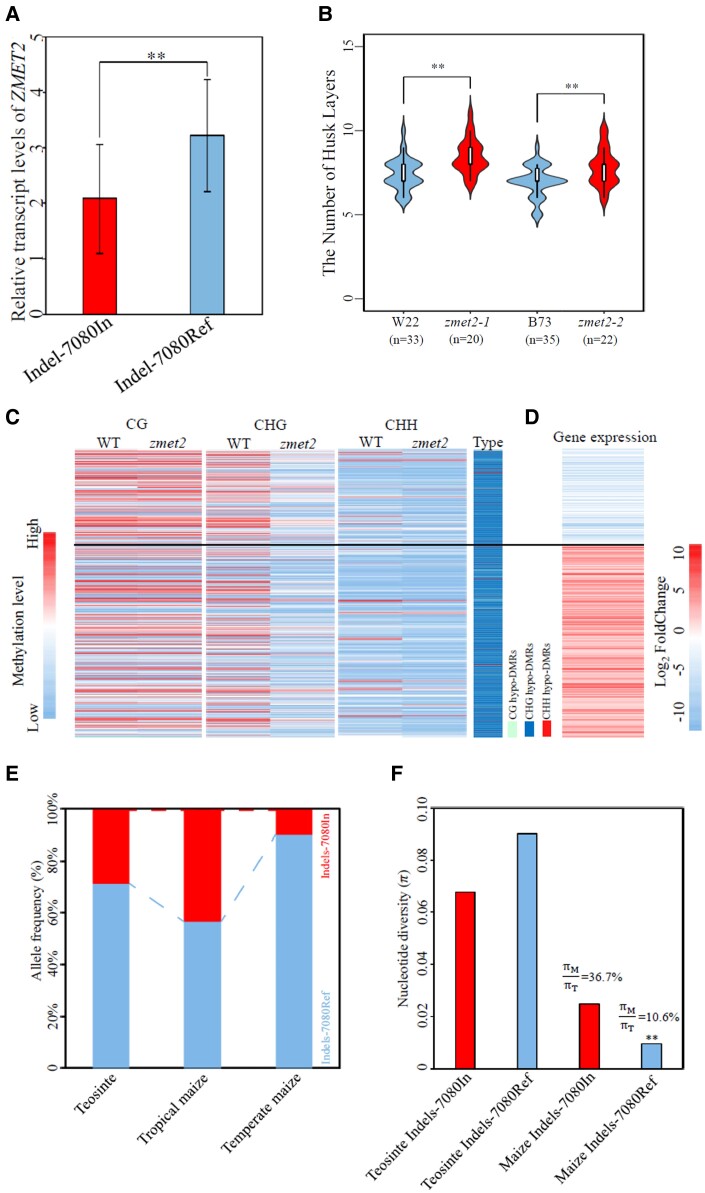
The 10-bp insertion in the 3′ UTR of *ZMET2* regulates its transcript level. *ZMET2* controls the husk layer in maize through regulating genome methylation, and the 10-bp insertion in its 3′ UTR is selected during the domestication from teosinte to maize. **A)** The relative transcript levels of *ZMET2* in maize inbred lines having the 10-bp insertion in the 3′UTR (Indel-7080In) and lines without the 10-bp insertion (Indel-7080Ref). **B)** The number of husk layers in *zmet* mutants (*zmet2-1* and *zmet2-2*) and their corresponding genetic backgrounds (W22 and B73, respectively). **C)** The differential methylation levels of genes in wild-type maize and the *zmet2* mutant. **D)** The differential expression of genes having differential methylation levels. **E)** The frequency of *ZMET2* alleles with the 10-bp insertion (Indels-7080In) and those without the insertion (Indels-7080Ref). **F)** Nucleotide diversity analysis of the region surrounding the 10-bp Indel in teosinte and maize. This figure is modified from Wang et al. 2024 (Wang et al. 2024).

The authors tested the effect of the 10-bp insertion on the transcript level of *ZMET2*. Expression study showed that maize lines without the 10-bp insertion had a higher transcript level of *ZMET2* compared to lines with the insertion (Fig. 1A) (Wang et al. 2024). The negative effect of the 10-bp insertion in the 3′ UTR on gene expression level was further demonstrated using luciferase as the reporter in maize mesophyll protoplast. The change of *ZMET2* transcript level hinted at the change of the genome methylation pattern. Sequence, expression, and genome methylation analyses suggested a positive correlation between *ZMET2* transcript level and both CHG and CHH methylations in the genome.

Using *zmet* mutants, Wang et al. confirmed that *ZMET2* decreased the number husk layers (Fig. 1B). The authors also tackled how *ZMET2* controls husk layer number through regulating genome methylation and gene expressions (Fig. 1, C and D). The results suggested that *ZMET2* promotes the methylation of genes related to biological processes, including telomere maintenance, anatomical structure homeostasis, and DNA geometric change, and thereby affects their expression levels. The results provide hints on genes for husk layer number regulation.

Maize is domesticated from its wild relative teosinte. It originated from a single domestication event in Mexico, a tropical region, but is now produced worldwide in various areas, including temperate regions (Matsuoka et al. 2002; Ranum et al. 2014). Tropical and temperate maize varieties that are adapted to different climates diverged 3,000 to 5,000 years ago (Li et al. 2017). The husk is an important structure for environmental adaptations. Compared to teosinte, the ear of maize is covered by more layers of husks, and tropical maize has more husk layers than temperate maize (Galinat 1975; Cui et al. 2016). Thus, Wang et al. also examined whether the 10-bp insertion in the 3′ UTR of *ZMET2* has been subjected to selection during domestication (Wang et al. 2024). The 10-bp insertion was found to be a genetic variant among teosinte accessions, and it is more abundant in tropical maize than temperate maize (Fig. 1E) (Wang et al. 2024). Further nucleotide diversity analysis revealed that the insertion had undergone intensive selection (Fig. 1F) (Wang et al. 2024).

In summary, the authors report the role of *ZMET2* in controlling the husk layer number in maize through regulating genome methylation. A 10-bp insertion in the 3′ UTR of *ZMET2* modulates its transcript level and the resulting genome methylation level. The study also revealed that the 10-bp insertion was selected during domestication of maize, and it is related to the adaptation of maize to tropical and temperate regions.
